# T330M Substitution in the Sodium-Dependent Phosphate Transporter NaPi2b Abolishes the Efficacy of Monoclonal Antibodies Against MX35 Epitope

**DOI:** 10.3390/antib14020030

**Published:** 2025-04-01

**Authors:** Leisan F. Bulatova, Vera S. Skripova, Aisylu R. Sagdeeva, Ramilia A. Vlasenkova, Tatiana A. Bugaenko, Rezeda R. Galimova, Alfiya I. Nesterova, Yuliya V. Filina, Ramziya G. Kiyamova

**Affiliations:** 1 Institute of Fundamental Medicine and Biology, Kazan Federal University, 420008 Kazan, Russia; bulatovalef@gmail.com (L.F.B.); v.s.skripova@yandex.ru (V.S.S.); ayrsagdeeva@kpfu.ru (A.R.S.); r.mukhamadeeva@yandex.ru (R.A.V.); tavady10@gmail.com (T.A.B.); jvfilina@kpfu.ru (Y.V.F.); 2 Republican Clinical Oncology Dispensary, 420029 Kazan, Russia; rezeda-galimova@mail.ru (R.R.G.); haalfy@mail.ru (A.I.N.)

**Keywords:** NaPi2b, *SLC34A2*, MX35 epitope, monoclonal antibodies, T330M mutation, targeted therapy, epitope recognition

## Abstract

**Background:** Monoclonal antibodies against the sodium-dependent phosphate transporter NaPi2b (*SLC34A2*) represent a promising approach in the treatment of ovarian and lung cancer. Of particular interest is the potential cancer-specific MX35 epitope of NaPi2b, as it serves as a target for monoclonal antibodies studied at various stages of preclinical and clinical trials. However, variations in the NaPi2b protein structure may limit the efficacy of therapeutic antibodies by affecting the accessibility of the MX35 epitope. **Methods:** An in silico analysis was performed using data from 101,562 tumor samples. Genomic DNA sequencing was conducted on blood samples from patients with ovarian carcinoma, breast cancer, and renal carcinoma to access the frequency of germline mutations in the *SLC34A2* gene region encoding the MX35 epitope. To assess the impact of the selected mutation, we generated a model cell line through site-directed mutagenesis carrying the mutant NaPi2b variant. **Results:** Using in silico analysis, we identified 17 unique variants in the *SLC34A2* gene leading to amino acid substitutions within the MX35 epitope of the NaPi2b. Among these, the most prevalent mutation, c.989C>T, resulting in p.T330M substitution, was detected in 5 out of 64 patients through genomic DNA sequencing. Using site-directed mutagenesis, we created the OVCAR-8/NaPi2b^p.T330M^ model cell line. L3 (28/1) monoclonal antibodies specific to the MX35 epitope failed to recognize the mutant NaPi2b^p.T330M^ variant compared to the wild-type of the NaPi2b in both Western blot and confocal microscopy experiments. **Conclusions:** The obtained data may serve as a basis for predicting the efficacy of monoclonal antibody-based targeted therapy binding to the MX35 epitope of NaPi2b in the treatment of oncological diseases.

## 1. Introduction

Cancer remains the leading cause of mortality worldwide, primarily due to late-stage diagnoses and the limited efficacy of current treatment strategies [1]. In the search for new therapeutic approaches, significant attention is being paid to targeted therapy, which is aimed at specific molecular targets in cancer cells. The efficacy and safety of targeted anticancer agents rely on their ability to selectively act on cancer cells rather than healthy tissues, which is determined by the nature and characteristics of their molecular targets.

The presence of germline or somatic mutations in genes encoding targets for targeted therapy can reduce the effectiveness of therapy by changing the conformation of the target and preventing its binding. At the same time, somatic mutations that arise in tumor cells during tumor evolution can either create new targets for targeted agents, contributing to the development of personalized therapy or, conversely, lead to drug resistance [2,3]. Germline mutations are associated with an increased risk of developing malignant neoplasms. For example, mutations in the *BRCA1* and *BRCA2* genes can significantly increase the likelihood of developing breast and/or ovarian cancer [4].

One promising target for targeted therapy is the membrane protein sodium-dependent phosphate transporter NaPi2b [5,6]. The NaPi2b transporter is localized on the surface of epithelial cells lining the intestine, type II alveolar cells, and epithelial cells from other organs and tissues, and it plays a crucial role in maintaining phosphate homeostasis in humans and other mammals [7,8,9].

The NaPi2b transporter has been proposed as a cancer biomarker [5,10,11], as high levels of NaPi2b have been detected in cells of ovarian, lung, and thyroid carcinomas, as well as in several other malignant neoplasms [7,12,13,14,15,16,17,18,19]. Currently, several antibody-based drugs targeting the NaPi2b transporter have been developed, including conjugates with cytotoxic compounds, which are at various stages of preclinical and clinical trials [20,21,22,23,24,25,26,27,28].

Therapeutic antibodies that target the MX35 epitope (311–340 aa) located in the NaPi2b transporter’s large extracellular domain (ECD, 188–361 aa) are of particular interest [5,21,22,29,30,31,32]. It has been shown that radiolabeled MX35-based antibodies, as well as their derivatives, when administered in vivo to humans or animals, predominantly accumulate in tumor foci and do not linger in healthy tissues and organs, even if they express NaPi2b on the cell surface [30,33].

According to previous findings from our laboratory, the in vitro accessibility of the MX35 epitope for antibody recognition depends on the presence of disulfide bonds that may form between cysteine residues C303, C322, C328, and C350, as well as glycosylation of asparagine residues N295, N308, N313, N321, N335, and N340 within the ECD [34,35]. We hypothesize that the MX35 epitope becomes more accessible for binding with antibodies in cancer cells due to a unique conformation of the ECD formed as a result of aberrant post-translational modifications, including N glycosylation and the formation of disulfide bonds, which may, in turn, be a consequence of malignant transformation [36,37,38,39,40,41]. This potential cancer-specific form of the MX35 epitope may provide high selectivity for targeted antitumor agents directed against it, thereby minimizing the risks of damage to healthy tissues and associated adverse side effects in patients that may arise from targeting other regions of the NaPi2b transporter.

However, the application of monoclonal antibodies against the MX35 epitope may be limited due to variations in the *SLC34A2* gene. It has been shown that amino acid substitution, specifically p.T330V [42], leads to a lack of recognition of the MX35 epitope by monoclonal antibodies. It is likely that other amino acid substitutions within the MX35 epitope of the NaPi2b transporter may also influence the accessibility of the MX35 epitope for antibody-based targeted therapy, rendering it ineffective in patients with such variations.

Therefore, investigating genetic variants in the *SLC34A2* gene, especially within the MX35 epitope region, in patients with malignant neoplasms is critical for identifying alterations that impact the recognition of the MX35 epitope by monoclonal antibodies. This is essential for optimizing the targeted application of monoclonal antibody therapy and refining personalized approaches to cancer treatment.

In this regard, this study aims to identify variants in the *SLC34A2* gene in patients with oncological diseases that lead to changes in the amino acid sequence within the MX35 epitope region of the NaPi2b transporter, which may affect its recognition by monoclonal antibodies.

## 2. Materials and Methods

### 2.1. Sample Collection and Genomic DNA Preparation for Sequencing

For this study, 64 peripheral blood samples were obtained from patients with ovarian carcinoma (*n* = 27), breast cancer (*n* = 20), and renal cell carcinoma (*n* = 17) undergoing treatment at the Republican Clinical Oncology Dispensary named after M.Z. Sigal in Kazan, Russia (RCOD). Additionally, 17 peripheral blood samples were collected from healthy volunteers without a significant oncological family history. Informed consent was obtained from all participants in the study. The collection, processing, and storage of biological material for the creation of a biobank were conducted with the approval of the local ethics committee of Kazan Federal University (KFU).

Genomic DNA was isolated from the collected peripheral blood samples using the GeneJET Whole Blood Genomic DNA Purification Mini Kit (Thermo Fisher Scientific, Waltham, MA, USA) according to the manufacturer’s protocol. The concentration of the isolated DNA was determined using a Qubit 4 fluorometer (Thermo Fisher Scientific, MA, USA) with the Qubit dsDNA HS Assay Kit (Thermo Fisher Scientific, MA, USA) following the manufacturer’s protocol.

### 2.2. Sequencing

The identification of variants in the *SLC34A2* gene was performed using whole-exome sequencing or Sanger sequencing on the genomic DNA samples obtained. For whole-exome sequencing, genomic DNA samples from 20 patients with triple-negative breast cancer and 17 patients with renal cancer were used. The control group comprised DNA samples from 8 healthy volunteers. DNA libraries were constructed using the SureSelectXT Human All Exon v8 kit (Agilent Technologies, Lexington, MA, USA) and sequenced on the NextSeq 500 platform (Illumina Inc., San Diego, CA, USA). Raw reads were aligned to the human reference genome hg38 using BWA v. 0.7.17 [43]. Enhancements to read alignment and whole-exome variant calling were performed using the Genome Analysis Toolkit (GATK) v.4 following GATK Best Practices recommendations [44]. For single nucleotide variant (SNV) calling, the Haplotype Caller module was employed. High-quality SNVs were filtered using vcf-annotate with parameters set to MinDP = 20, Qual = 10, and MinMQ = 10, and subsequently annotated using wANNOVAR [45].

Sanger sequencing was performed by Evrogen JSC using a 3500xL Applied Biosystems genetic analyzer (Thermo Fisher Scientific, MA, USA). The target region within the *SLC34A2* gene was analyzed in samples prepared from the genomic DNA of peripheral blood cells from ovarian carcinoma patients (*n* = 27) and healthy women (*n* = 9).

For this purpose, the *SLC34A2* gene region encoding the ECD, which contains the MX35 epitope of the NaPi2b (g.15415–16250; *SLC34A2* sequence NG_021185.2), was amplified using PCR using primers SLC34A2_For (5′-GAGCATTCATGCCCTCTGT-3′) and SLC34A2_Rev (5′-CAAGTGACCAGCCCATCTC-3′). Primer sequences were selected using online tools, such as Primer-BLAST (integrated into the NCBI database) and PrimerQuest Tool (Integrated DNA Technologies, Inc., Coralville, IA, USA), and were custom-synthesized by Evrogen JSC (Moscow, Russia). PCR was carried out using the Tersus Plus PCR kit (Evrogen JSC, Moscow, Russia). Amplification products were purified from the reaction mixture using the Cleanup S-Cap kit (Evrogen JSC, Moscow, Russia), and reverse primer SLC34A2_Rev Sanger sequencing of the obtained PCR products was performed with the reverse primer SLC34A2_Rev.

### 2.3. Cell Lines and Bacterial Strains

In this study, the human ovarian carcinoma cell line OVCAR-8 (Creative Biolabs, New York, NY, USA) was used. We have previously shown that OVCAR-8 cells lack endogenous expression of NaPi2b [35]. The cells were cultured under standard protocol in complete RPMI-1640 medium (Paneco, Moscow, Russia) supplemented with 10% fetal bovine serum (Capricorn, Ebsdorfergrund, Germany), 2 mM alanyl-glutamine, 100 U/mL penicillin, and 100 µg/mL streptomycin (Paneco, Moscow, Russia).

To generate recombinant plasmids encoding the NaPi2b transporter with the p.T330M amino acid substitution, chemically competent *E. coli* XL1-Blue cells (Evrogen JSC, Moscow, Russia) were used. The bacterial cells were cultured in an LB lysogeny medium (AppliChem, Darmstadt, Germany).

### 2.4. Site-Directed Mutagenesis

To generate the recombinant plasmid pcDNA3.1(+)/NaPi2b-T330M encoding the missense variant of the NaPi2b transporter with p.T330M substitution, site-directed mutagenesis was performed using the Quik Change II XL Site-Directed Mutagenesis Kit (Agilent, MA, USA) according to the manufacturer’s instructions. The previously constructed recombinant plasmid pcDNA3.1(+)/NaPi2b-WT containing the wild-type NaPi2b phosphate transporter cDNA (*SLC34A2*, NM_006424.2) [42] was used as a template. The following primers were used for site-directed mutagenesis: T330M_For (5′-CCTTCCCTCTGTTGGATGGATGGC-3′) and T330M_Rev (5′- GCCATCCATCCAACAGAGGGAAGG-3′). The presence of the genetic variants was confirmed using Sanger sequencing with the primer SLC34A2_Seq (5′-GGTGCTCTTGCCCGTGG-3′).

### 2.5. Transfection

Transfection was conducted with the JetPRIME transfection reagent (Polyplus, New York, NY, USA) according to the manufacturer’s instructions. Twenty-four hours post-transfection, the cells were used to prepare samples for Western blot analysis or microscopy. OVCAR-8/NaPi2b^p.T330M^ cells with the pcDNA3.1(+)/NaPi2b-T330M plasmid were used to study the effect of the p.T330M substitution on the recognition of the MX35 epitope by monoclonal antibodies. OVCAR-8/NaPi2b^WT^ cells with the pcDNA3.1(+)/NaPi2b-WT plasmid encoding the wild-type NaPi2b transporter were used as a positive control. OVCAR-8/pcDNA3.1(+) cells with the empty pcDNA3.1(+) plasmid were used as a negative control.

### 2.6. Western Blot Analysis

For Western blot analysis, protein lysates were prepared from OVCAR-8/pcDNA3.1(+), OVCAR-8/NaPi2b^WT^, and OVCAR-8/NaPi2b^p.T330M^ cells using RIPA buffer (Thermo Fisher Scientific, MA, USA) supplemented with a protease and phosphatase inhibitor cocktail (Thermo Fisher Scientific, MA, USA). Proteins were separated under denaturing conditions in a 10% polyacrylamide gel using the MINI-Protean TETRA system (Bio-Rad, Hercules, CA, USA) following the manufacturer’s protocol. Protein transfer from the gel to a PVDF membrane was performed via wet transfer using a transfer buffer (0.25 M Tris base, 1.92 M glycine, 20% methanol, pH 8.3).

To detect the MX35 epitope of the NaPi2b transporter, primary mouse monoclonal antibodies L3(28/1) [46] were utilized. To assess the level of NaPi2b protein in the samples, primary mouse monoclonal antibodies N-NaPi2b (15/1), targeting the intracellular N-terminal domain of NaPi2b (1–100 aa) and unaffected by the structure changes in the MX35 epitope region, were employed [47]. For loading control, primary mouse monoclonal antibodies against glyceraldehyde-3-phosphate dehydrogenase (GAPDH; HyTest, Moscow, Russia) were used.

Goat anti-mouse IgG secondary antibodies conjugated with horseradish peroxidase (Thermo Fisher Scientific, MA, USA) were used for detection. Chemiluminescent signals were visualized using the ChemiDoc XRS+ imaging system (Bio-Rad, CA, USA) and Image Lab 6.0.1 software (Bio-Rad, CA, USA). All experiments were performed at least in three independent biological replicates. Western blot data were statistically analyzed using an unpaired Student’s *t*-test.

### 2.7. Laser Confocal Microscopy

The effect of the p.T330M substitution in the NaPi2b transporter on the recognition of the MX35 epitope by monoclonal antibodies was analyzed using laser confocal microscopy on fixed and permeabilized OVCAR-8/NaPi2b^WT^ and OVCAR-8/NaPi2b^p.T330M^ cells. Two days before the experiment, cells were seeded onto glass-bottom culture dishes at a density of 75,000 cells per dish (Mattek, Ashland, MA, USA). One day before the experiment, the cells were transfected with the corresponding plasmids, as described in the Section 2.5.

Cell fixation was performed using a 4% paraformaldehyde solution (AppliChem, Darmstadt, Germany) for 20 min at room temperature. Blocking and permeabilization were carried out using a blocking buffer (3% bovine serum albumin in phosphate-buffered saline containing 0.1% Triton X-100; AppliChem, Darmstadt, Germany) for 1 h at room temperature. The cells were sequentially stained with primary monoclonal antibodies L3(28/1) and N-NaPi2b (15/1), and secondary Goat anti-mouse IgG antibodies conjugated with Alexa Fluor 488 (Thermo Fisher Scientific, MA, USA) in blocking buffer. For nuclear visualization, Hoechst 33,342 dye (Sigma Aldrich, MA, USA) was applied for 10 min at room temperature. Visualization of the samples was conducted using a LSM700 laser scanning confocal microscope (Zeiss, Oberkochen, Germany).

### 2.8. Bioinformatic Analysis of Public Databases

To identify variants in the *SLC34A2* gene that alter the amino acid sequence in the MX35 epitope region of the NaPi2b transporter in patients with various oncological diseases, whole-exome sequencing data from tumor tissue genomic DNA were analyzed using public databases such as cBioPortal [48], The International Cancer Genome Consortium (ICGC) [49], and the Catalogue of Somatic Mutations in Cancer (COSMIC) [50]. The cBioPortal database provided access to the results of 32 recent studies from The Cancer Genome Atlas (TCGA, *n* = 10,967) and 33 studies from the AACR Project Genie (*n* = 85,372). Additionally, results from 14 studies in the ICGC database (*n* = 1868) and 34 studies in the COSMIC database (*n* = 3355) were extracted.

To predict the functional significance of identified variants, they were analyzed using the following in silico tools, which were employed to analyze missense variants due to gathering diverse information and improve the accuracy of predicting the negative effects of mutations: PROVEAN, SIFT, PolyPhen-2, Panther-PSEP, FATHMM, and Mutation Assessor [51,52]. Variants that disrupted the amino acid sequence via stop codon formation, frameshifts, or splicing disruptions were also deemed functionally significant due to their substantial impact on the transcription and translation.

The prevalence of identified variants in the population was evaluated using the minor allele frequency (MAF) metric derived from the gnomAD (The Genome Aggregation Database) [53] for the general population and RUSeq for the Russian population [54].

The MX35 epitope of the NaPi2b model was obtained from the AlphaFold Protein Structure Database [55]. The AlphaFoldDB ID for this model is AF-O95436-F1. The visualization of the MX35 epitope of NaPi2b was performed using the Mol* (molstar) tool (https://molstar.org/) [56].

## 3. Results

### 3.1. Identification of Variations in the MX35 Epitope of the Sodium-Dependent Phosphate Transporter NaPi2b

**Figure 1:**
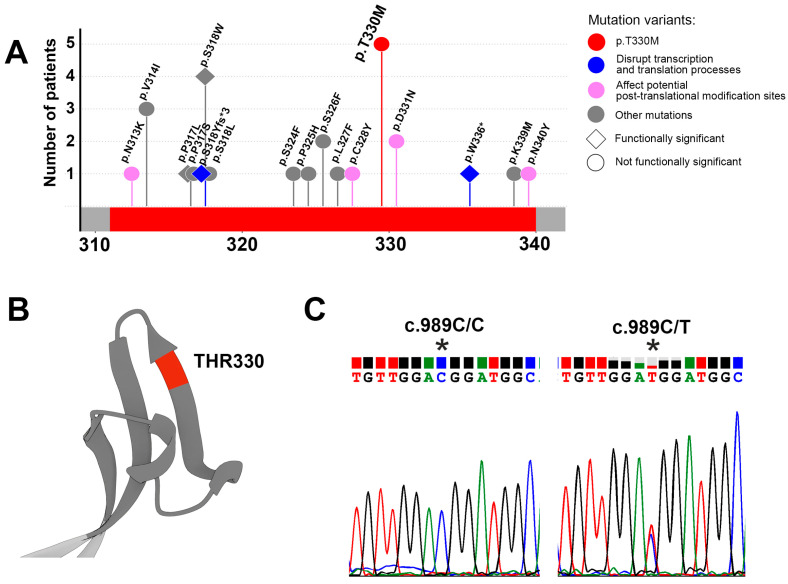
Identification of the p.T330M substitution in the MX35 epitope region of the sodium-dependent phosphate transporter NaPi2b. (**A**) Schematic representation of NaPi2b structure, indicating the part of ECD (grey) and MX35 epitope region (red) with variations in the MX35 epitope region identified through in silico analysis. (**B**) A model of the MX35 epitope of NaPi2b, based on AlphaFold predictions (identifier: AF-O95436-F1), highlights the threonine residue at position 330. (**C**) A representative chromatogram of the *SLC34A2* gene region encoding the MX35 epitope of NaPi2b illustrates the c.989C/C (wild-type, WT) and c.989C/T (p.T330M) sequences. * Denotes the target nucleotide at position c.989 of the *SLC34A2* gene, the mutation of which results in the amino acid substitution p.T330M in the NaPi2b protein.

Tumor tissue genomic DNA sequencing data were obtained for 101,562 patients with various oncological diseases from open databases including cBioPortal (TCGA and AACR Project Genie), ICGC, and COSMIC. The patients were categorized into 27 groups based on tumor localization (Supplementary Table S1). Seventeen unique variants in the *SLC34A2* gene leading to alterations in the amino acid sequence within the MX35 epitope (311–340 aa) of the NaPi2b transporter were identified (Figure 1A). Variations within the MX35 epitope of the NaPi2b transporter were detected in 13 out of 27 tumor groups, including tumors in the bone marrow, breast, central nervous system, connective tissue, esophagus and stomach, intestine, kidney, lung, lymph node, prostate gland, skin, thyroid gland, and of unknown origin. (Supplementary Table S2).

Out of 17 variants, 4 were classified as functionally significant (p.P317L, p.S318W, p.S318Yfs*3, p.W336*); 2 variants disrupted transcription and translation processes (p.S318Yfs*3, p.W336*, blue color); while four affected potential N-glycosylation sites and cysteine residues that could form disulfide bonds (p.N313K, p.C328Y, p.D331N, p.N340Y, purple color) (Figure 1A). All identified variants in the MX35 epitope region of the NaPi2b transporter exhibited a low (<1%) prevalence in the general population according to gnomAD data (Supplementary Table S2).

Among the identified variants, the prevalence of the c.989C>T resulting in amino acid substitution p.T330M was higher than that of the other variants. This variant was not predicted to be functionally significant; however, it is noteworthy that its frequency in the Russian Federation appears to be higher (0.012) than in the general population according to the GnomAD database (0.008).

The threonine residue at position 330 of the NaPi2b contributes to forming a beta-structure and is located in the middle of the MX35 epitope. This epitope, in turn, forms a loop within the ECD, as depicted in the AlphaFold structure (Figure 1B).

### 3.2. Identification of Germline Variants in the MX35 Epitope of the Sodium-Dependent Phosphate Transporter NaPi2b

To identify variants in the *SLC34A2* gene, which encodes the NaPi2b transporter, genomic DNA sequencing was performed on samples obtained from the peripheral blood of 64 patients with malignant neoplasms (ovarian carcinoma (*n* = 27), breast cancer (*n* = 20), and renal cell carcinoma (*n* = 17)) receiving treatment at the Republican Clinical Oncology Dispensary named after M.Z. Sigal in Kazan, Russia. This was accompanied by a control group of 17 healthy donors without a significant oncological family history. The characteristics of the studied cohort are presented in Table 1.

The sequencing results revealed only one variant in the *SLC34A2* gene region, encoding the MX35 epitope region: a heterozygous substitution c.989C>T leading to the amino acid change p.T330M of the NaPi2b transporter. The C.989C>T variant was detected in 5 out of 64 (7.8%) patients (Figure 1C, Table 1). Among these patients, there were three with kidney cancer, one with ovarian cancer, and one with breast cancer. No variants resulting in amino acid substitutions in the MX35 epitope of the NaPi2b transporter were identified in the control group of healthy volunteers.

### 3.3. The Impact of the Amino Acid Substitution P.T330M on the Recognition of the MX35 Epitope by Monoclonal Antibodies in Ovarian Carcinoma Cells

**Figure 2:**
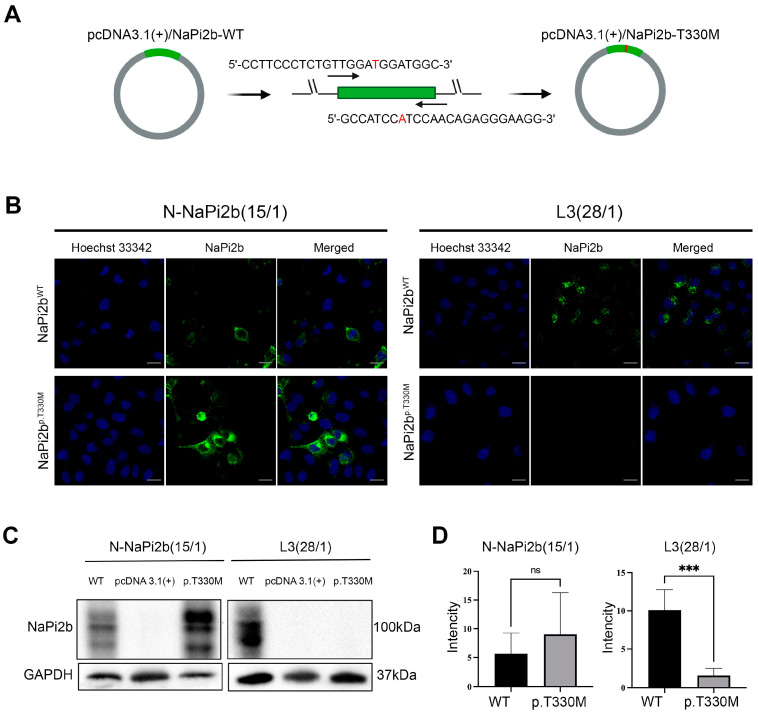
Recognition of the MX35 epitope by monoclonal antibodies in the mutant form of the sodium-dependent phosphate transporter NaPi2b with the p.T330M substitution. (**A**) Generation of the mutant form p.T330M of NaPi2b transporter through site-directed mutagenesis of the pcDNA3.1(+) expression vector encoding *SLC34A2* gene. (**B**) Confocal microscopy images show the localization of NaPi2b in OVCAR-8 cells expressing either wild-type (WT) or mutant (T330M) NaPi2b, using monoclonal antibodies targeting the MX35 epitope (L3(28/1)) or the N-terminal domain (N-NaPi2b(15/1)); scalebar is 20 µm. (**C**) Western blot analysis of NaPi2b expression in OVCAR-8 cells, expressing either empty pcDNA 3.1(+) vector, or wild-type (WT) or mutant (p.T330M) form of the NaPi2b. (**D**) Quantitative analysis of NaPi2b signal intensity obtained using N-NaPi2b(15/1) (**left**) and L3(28/1) (**right**) antibodies based on Western blot data. Results are presented as mean ± SD. Statistical significance is indicated as *** (*p* < 0.001), ns is not significant.

The next step involved investigating the impact of the amino acid substitution p.T330M of the NaPi2b transporter on the recognition of the MX35 epitope by monoclonal antibodies in vitro using the human ovarian carcinoma cell line OVCAR-8, which transiently expressed either the mutant form (NaPi2b^p.T330M^) or the wild-type (NaPi2b^WT^) transporter (Figure 2A). The availability of the MX35 epitope was assessed through confocal microscopy and Western blot analysis using monoclonal antibodies L3(28/1) [46].

According to the results obtained using confocal microscopy, antibodies L3(28/1) against the MX35 epitope bind exclusively to the wild-type NaPi2b transporter and not to its mutant form NaPi2b^p.T330M^ in OVCAR-8/NaPi2b^WT^ and OVCAR-8/NaPi2b^p.T330M^ cells, respectively (Figure 2B). The presence of recombinant forms of NaPi2b^p.T330M^ and NaPi2b^WT^ in the analyzed samples was confirmed by N-NaPi2b (15/1) antibodies, which target its N-terminal domain (1–100 aa); this recognition is independent of amino acid substitutions in the extracellular domain.

The results from the Western blot analysis corroborated the findings obtained via confocal microscopy (Figure 2C). The specific binding of antibodies L3(28/1) to the MX35 epitope was observed in OVCAR-8/NaPi2b^WT^ cell lysates, whereas binding to OVCAR-8/NaPi2b^p.T330M^ cell lysates was significantly lower (Figure 2D). Antibodies N-NaPi2b (15/1) recognized the intracellular N-terminal domain of the NaPi2b transporter in all examined samples, confirming the presence of both forms of NaPi2b (NaPi2b^p.T330M^ and NaPi2b^WT^) in the studied cells (Figure 2C,D).

Thus, using Western blot analysis and confocal microscopy, we demonstrated that monoclonal antibodies L3(28/1) do not recognize NaPi2b with p.T330M substitution within the MX35 epitope.

## 4. Discussion

Monoclonal antibody-based drugs are increasingly employed for the treatment of various diseases, including cancer [57]. However, alterations in the amino acid sequence or a reduction in the expression levels of target proteins in cancer cells are among the primary causes of diminished efficacy of antibody-based targeted therapies [58].

The sodium-dependent phosphate transporter NaPi2b is a highly promising target for targeted cancer treatment due to overexpression in the range of tumors and potential cancer-specific epitope MX35. A range of therapeutic agents based on humanized monoclonal antibodies has been developed against NaPi2b, and these agents, which are currently at various stages of preclinical and clinical trials, have shown considerable promise for cancer treatment. Among these are drugs [21,22,29,30,31,32] targeting the potential cancer-specific epitope MX35 (311–340 aa) of the NaPi2b transporter. We hypothesize that this epitope becomes more accessible for antibody recognition specifically in cancer cells, which could provide high specificity and safety for targeted drugs aimed at it.

Previously, it was demonstrated that the MX35 epitope is not recognized by monoclonal antibodies when the p.T330V amino acid substitution occurs in the NaPi2b, which may serve as a contraindication for the use of antibody-based targeted therapies directed at this epitope [42].

Consequently, the identification of variants in the *SLC34A2* gene encoding the NaPi2b transporter that affect the recognition of the MX35 epitope by monoclonal antibodies is crucial for predicting the efficacy and determining the appropriateness of prescribing antibody-based targeted therapies to patients.

The in silico analysis of 101,562 tumor samples performed during this study revealed 17 unique variants in the *SLC34A2* gene leading to alterations in the amino acid sequence within the MX35 epitope (311–340 aa) of the NaPi2b transporter. Among them, 4 out of 17 mutations were classified as functionally significant, including p.P317L, p.S318W, p.S318Yfs*3, and p.W336*. Variations within the MX35 epitope of the NaPi2b transporter were detected in 13 out of 27 tumor groups, including tumors in the bone marrow, breast, central nervous system, connective tissue, esophagus and stomach, intestine, kidney, lung, lymph node, prostate gland, skin, thyroid gland, and of unknown origin.

In this study, genomic DNA sequencing was conducted on 64 patients with malignant tumors. The results revealed a single nucleotide variant, c.989C>T, leading to the amino acid substitution p.T330M in 5 patients. Considering previously published data on the impact of the threonine-to-valine substitution (p.T330V) on the recognition of the MX35 epitope by monoclonal antibodies [42], along with our sequencing data, we decided to investigate how the p.T330M variant affects MX35 epitope recognition in NaPi2b. It was shown that L3(28/1) antibodies, directed to the MX35 epitope, fail to recognize the NaPi2b p.T330M mutant form.

The results obtained indicate that patients with the p.T330M as well as p.T330V mutation will be insensitive to therapy with antibodies directed towards the MX35 epitope. The molecular mechanism of the p.T330M mutation and how it impacts antibody binding remain unclear and still need to be elucidated. We can only speculate that the substitution of threonine with methionine at position 330 may disrupt the protein’s conformation (e.g., alter the shape of the epitope), thereby affecting antibody binding by changing the charge (replacing a polar amino acid (T) with a non-polar one (M)) [42]. The assessment of the interaction of monoclonal antibodies with the MX35 epitope using current methods, including Hydrogen–Deuterium Exchange Mass Spectrometry (HDX-MS), remains difficult [59]. This is due to the complexities involved in studying the structure and conformational dynamics of membrane proteins, which limits the ability to obtain detailed information on the mechanisms of antibody binding.

To date, several mutations have been described in the NaPi2b transporter gene *SLC34A2* [60]. Disruption of the functional activity of the NaPi2b transporter, caused by pathogenic variants in the *SLC34A2* gene, is considered a contributing factor to a rare autosomal recessive disease known as pulmonary alveolar microlithiasis (PAM), characterized by the accumulation of concretions, primarily composed of calcium phosphates in the alveoli, leading to lung tissue damage [61]. Currently, no published data exist on the association of the 17 mutations we identified in the MX35 epitope region with any diseases, including pulmonary alveolar microlithiasis (PAM). It should be noted that the c.989C>T (p.T330M) in the *SLC34A2* gene was described in the control group of healthy donors but not in patients with PAM [62]. Since this variant is located outside the co-transporter domains of NaPi2b, the in silico analysis conducted in this study did not indicate that the amino acid substitution p.T330M is functionally significant or affects the transport activity of NaPi2b, which is associated with PAM development.

A significant limitation of the current study is that the presence of the p.T330M mutation in a patient does not imply that all therapeutic antibodies targeting NaPi2b will be ineffective. This may apply to novel humanized monoclonal antibodies currently undergoing clinical and preclinical trials which can target a different region of the NaPi2b than the MX35 epitope [23,24,25,26,27,28].

The results of this and future studies should be taken into consideration when prescribing targeted drugs aimed at the MX35 epitope to improve the efficacy of treatment and the quality of life for patients with malignant tumors.

Since the mutant form of NaPi2b with the p.T330M substitution was generated under in vitro conditions, it is essential to confirm these findings through in vivo experiments. Specifically, it would be important to study the interaction of monoclonal antibodies with NaPi2b expressed in tumors from patients carrying germline or somatic p.T330M substitutions.

## 5. Conclusions

In this study, a database analysis identified 17 variants located in the *SLC34A2* region encoding the MX35 epitope of the large extracellular domain of the sodium-dependent phosphate transporter NaPi2b in several malignant tumors, including p.T330M substitution. Heterozygous variant c.989C>T (p.T330M) was also detected in blood genomic DNA samples in 5 out of 64 patients with breast, kidney, and ovarian cancer.

Experimental evidence demonstrated that the p.T330M substitution results in a drastic decline in the ability of L3(28/1) monoclonal antibodies to recognize the MX35 epitope, thereby limiting the applicability of monoclonal antibody-based targeted therapy directed to MX35 epitope for patients harboring this variant. In the future, we plan to generate new monoclonal antibodies and test their ability to bind wild-type and the p.T330M mutant form of NaPi2b, as well as expand our studies by focusing on identifying mutations in the NaPi2b transporter in cancer patients beyond the MX35 epitope. The data we obtained are important for developing more effective strategies for targeted therapy of oncological diseases and understanding the molecular mechanisms of antigen–antibody interactions, including therapeutic antibodies.

## Figures and Tables

**Table 1:** Clinical characteristics and genomic DNA sequencing results of patients with malignant neoplasms treated at the Republican Clinical Oncology Dispensary named after M.Z. Sigal, along with data from the control group.

Diagnosis	Genotype			Total
	Wild Type c.989C/C	Heterozygous c.989C/T	Homozygous c.989T/T	
Breast cancer	19	1	0	20
Kidney cancer	14	3	0	17
Ovarian cancer	26	1	0	27
Healthy (control)	17	0	0	17

## Data Availability

The original contributions presented in this study are included in the article/Supplementary Materials. Further inquiries can be directed to the corresponding author.

## Supplementary Materials

The following supporting information can be downloaded at: https://www.mdpi.com/article/10.3390/antib14020030/s1, Table S1: Characteristics of tumor tissue samples from open databases (cBioPortal, TCGA, AACR Project Genie), ICGC, and COSMIC grouped by tumor localization used to identify mutations in the *SLC34A2* gene region encoding the MX35 epitope of the sodium-dependent phosphate transporter NaPi2b; Table S2: List of mutations in the *SLC34A2* gene altering the amino acid sequence within the MX35 epitope region (311–340 aa) of the sodium-dependent phosphate transporter NaPi2b, identified through whole-exome sequencing data from cBioPortal (TCGA, AACR Project Genie), ICGC, and COSMIC.
