# Supplementary material for: T330M Substitution in the Sodium-Dependent Phosphate Transporter NaPi2b Abolishes the Efficacy of Monoclonal Antibodies Against MX35 Epitope
Source: Antibodies (Basel). 2025 Apr 1;14(2):30. doi: 10.3390/antib14020030 (PMC12015770; doi:10.3390/antib14020030)
Supplement: Supplementary file 1 [file antibodies-14-00030-s001.zip › antibodies-3511629-supplementary.pdf]

Supplementary.

Table S1. Characteristics of tumor tissue samples from open databases (cBioPortal, TCGA, AACR Project Genie), ICGC, and COSMIC grouped by tumor localization used to identify mutations in the *SLC34A2* gene region encoding the MX35 epitope of the sodium-dependent phosphate transporter NaPi2b.

№	Tumor Localization	Number of Samples Analysed				Total number of samples
		cBioPortal, TCGA, 32 studies	cBioPortal, AACR Project Genie, 33 studies	ICGC, 14 studies	COSMIC, 34 studies	
1	Adrenal gland	92	-	-	-	92
2	Biliary Tract	36	-	12	15	63
3	Bladder	411	2118	-	50	2579
4	Bone	-	718	61	-	779
5	Bone marrow	200	2820	98	-	3118
6	Breast	1084	11742	120	-	12946
7	Central nervous system	1106	6609	217	23	7955
8	Cervix	297	-	-	-	297
9	Connective tissue	433	2752	-	20	3205
10	Esophagus and stomach	622	4419	107	-	5148
11	Eye	80	-	-	-	80
12	Head and Neck	523	2223	13	111	2870
13	Intestine	594	9682	-	863	11139
14	Kidney	860	1665	74	204	2803
15	Liver	372	-	293	-	665
16	Lung	1053	14844	66	763	16726
17	Lymph node	48	2625	100	-	2773
18	Ovary	585	3527	69	15	4196
19	Pancreas	184	3613	314	-	4111
20	Pleura	87	620	-	-	707
21	Prostate gland	494	3490	254	-	4238
22	Skin	448	4537	70	1238	6293
23	Testis	149	-	-	-	149
24	Thymus	123	-	-	-	123
25	Thyroid gland	500	1398	-	23	1921
26	Unknown origin	-	3065	-	-	3065
27	Uterus	586	2905	-	30	3521
Total number of samples		10967	85372	1868	3355	101562

Table S2. List of mutations in the *SLC34A2* gene altering the amino acid sequence within the MX35 epitope region (311-340 aa) of the sodium-dependent phosphate transporter NaPi2b, identified through whole-exome sequencing data from cBioPortal (TCGA, AACR Project Genie), ICGC, and COSMIC.

№	Amino Acid Substitution in ECD NaPi2b	MAF Frequency		Functionally Significant	Sample, Source and Localization
		gnomAD	RUSeq		
1	p.N313K	-	-	No	cBioPortal, Genie:<br>1/11742 - Breast
2	p.V314I	0,000058240	-	No	cBioPortal, Genie:<br>1/2752 - Connective tissue<br>1/6609 - Central nervous system
					cBioPortal, TCGA:<br>1/494 - Prostate gland
3	p.P317L	-	-	Yes	cBioPortal, Genie:<br>1/2625 - Lymph node
4	p.P317S	-	-	No	cBioPortal, Genie:<br>1/4537 - Skin
5	p.S318L	0,000001859	-	No	cBioPortal, Genie:<br>1/1398 - Thyroid gland
6	p.S318W	0,000002478	-	Yes	cBioPortal, Genie:<br>2/11742 - Breast<br>2/1665 - Kidney
7	p.S318Yfs*3	-	-	Yes	COSMIC:<br>1/1238 - Skin
8	p.S324F	0,000002478	-	No	COSMIC:<br>1/1238 - Skin
9	p.P325H	-	-	No	ICGC:<br>1/107 - Esophagus and stomach
10	p.S326F	-	-	No	COSMIC:<br>2/1238 - Skin
11	p.L327F	-	-	No	cBioPortal, TCGA:<br>1/448 - Skin
12	p.C328Y	-	-	No	COSMIC:<br>1/763 - Lung
13	p.T330M	0,008124000	0,01155	No	ICGC:<br>2/98 - Bone marrow<br>2/66 - Lung
					COSMIC:<br>1/23 - Thyroid gland
14	p.D331N	-	-	No	cBioPortal, TCGA:<br>1/448 - Skin
					cBioPortal, Genie:<br>1/3065 - Unknown origin
15	p.W336*	-	-	Yes	cBioPortal, TCGA:<br>1/448 - Skin
16	p.K339M	-	-	No	ICGC:<br>1/107 - Esophagus and stomach
17	p.N340Y	-	-	No	COSMIC:<br>1/863 - Intestine
